# Impact of a fumaric acid and palm oil additive on beef cattle performance and carcass characteristics in diets containing increasing concentrations of corn silage^1^

**DOI:** 10.1093/tas/txaa043

**Published:** 2020-04-08

**Authors:** Hannah Carissa Wilson, Fred H Hilscher, Levi J McPhillips, Bradley M Boyd, Andrea K Watson, Galen E Erickson, Jim C MacDonald

**Affiliations:** Department of Animal Science, University of Nebraska, Lincoln, NE

**Keywords:** cattle, corn silage, essential oils, performance

## Abstract

A feedlot study was conducted comparing a natural feed additive at varying corn silage (CS) inclusions on receiving and finishing cattle performance. The study utilized 480 crossbred steers (initial shrunk body weight [BW] = 296 kg; SD = 24.1 kg) in 48 pens with 10 steers/pen and 8 pens per treatment. Treatments were designed as a 2 × 3 factorial with 3 inclusions of CS (14%, 47%, 80%; dry matter [DM] basis) with or without (+, −) the inclusion of a feed additive containing fumaric acid and palm oil (FAPO). All treatment diets contained 16% modified distillers grains plus solubles and 4% supplement with dry-rolled corn replacing CS on a DM basis. All steers were fed the 80 CS diet and adapted to 47% and 14% CS over a 10- and 24-d period, respectively. Cattle fed 80 CS were fed for 238 days, 47 CS for 195 days, and 14% CS were fed for 168 days to a common backfat of 1.28 cm (*P* ≥ 0.59). There were no interactions for CS inclusion and the inclusion of FAPO on final body weight (FBW), DMI, ADG, G:F, hot carcass weight (HCW), LM area, marbling, or calculated yield grade (CYG; *P* ≥ 0.15). There was no significant difference for FBW, DMI, ADG, G:F, HCW, marbling, or CYG for cattle fed with or without FAPO (*P* ≥ 0.13). However, there was a quadratic response for FBW, ADG, G:F, HCW, marbling, and CYG with increased inclusion of CS (*P* ≤ 0.04). Inclusion of FAPO had no effect on performance. Feeding CS at greater inclusions decreased daily gain and feed efficiency but increased FBW when fed to an equal fat endpoint. CS gave greater returns ($/animal) when fed at 80% of diet DM. Feeding greater amounts of CS can be an economical way to finish cattle. In this study, FAPO did not affect animal performance, carcass characteristics, or economic return.

## INTRODUCTION

Essential oils have grown as a popular additive to prevent ruminal acidosis, bloat, and digestive and metabolic upsets (Nocek 1997; Benchaar et al. 2006b). Essential oils (EO) are aromatic, volatile liquids obtained from plant material through steam distillation, and named after the plant from which they are derived. Palm oil is obtained from *Elaeis guineensis* and contains mainly mono-, di-, and triglycerides and is rich in vitamin E. Various mixtures of EO have antimicrobial properties against a wide range of microorganisms found in the rumen of cattle including bacteria, fungi, viruses, and protozoa which has been associated with altered ruminal metabolism, improve feed efficiency and animal productivity (Greathead 2003; Benchaar et al. 2008). Early studies found inhibition of ruminal bacteria activity *in vitro*, but the degree of inhibition was dependent on the chemical structure of the EO compound (Oh et al. 1967; Nagy and Tengerdy 1968). In most studies, despite which EO was used, researchers have found a decrease, or no change, in total VFA concentration but found a shift in molar proportions in favor of propionate (McGuffey et al. 2001; Benchaar et al. 2008). However, effects of plant extracts on ruminal microbial fermentation are pH dependent (Skandamis and Nychas 2000; Cardozo et al. 2005). Therefore, ruminal pH could be an important factor when evaluating EO supplementation in various inclusions of concentrate.

Between 2% and 12% of energy is lost in the form of methane in ruminants (Johnson and Johnson 1995). This energetic loss is less in high concentrate diets due to less available hydrogens for methanogenesis, primarily due to increased propionate production (Shabat et al. 2016). Intermediates of the propionate–succinate pathway (specifically fumaric acid) have been suggested as rumen manipulators to increase propionate production and increase performance (Li et al. 2010; Yang et al. 2012). However, other authors reported no increase in propionate (De Nardi et al. 2014; Vyas et al. 2015). Thus, these discrepancies have been attributed to diet-dependent defects. Several studies evaluated the effects of fumaric acid in both high concentrate and high forage diets and reported that fumaric acid led to greater responses in high forage diet compared with high concentrate diets, attributing differences in dissolved hydrogen concentrations and microbiota populations ultimately affecting conversion of fumaric acid to propionate (García-Martínez et al. 2005; Yang et al. 2012; Li et al. 2018).

As corn silage (CS) is added to a diet replacing corn grain, energy density decreases and is less available for body weight (BW) gain (Hilscher et al. 2019). Added CS also alters ruminal pH by changing the amount of highly fermentable starch available in the rumen (Galyean and Defoor 2003). CS is a valuable tool to mimic different stages of production (growing and finishing) to assess the efficacy of feed additives at different concentrate levels. Very few studies have been performed to evaluate the effects of EO, and more specifically an additive containing fumaric acid and palm oil (FAPO), on beef cattle. We hypothesized that FAPO may interact with the forage to concentrate ratio and affect animal performance. The objective of this study was to determine the impact of a fumaric acid and palm additive on the performance and carcass characteristics of beef cattle fed at different inclusions of CS.

## MATERIALS AND METHODS

A finishing experiment conducted at the Eastern Nebraska Research and Extension Center utilized 480 crossbred steers (initial shrunk BW 296 ± 24.1 kg). Cattle were limit fed a common diet, consisting of 1/3 sweet bran (Cargill Corn Milling, Blair, NE), 1/3 alfalfa hay, and 1/3 dry-rolled corn at 2% of BW for 5 d before the start of the experiment. Two-day initial weights were recorded on d 0 and 1 which were averaged and used as the initial BW. Cattle were weighed individually using a hydraulic squeeze chute (Silencer, Dubas Equipment, NE) prior to feeding.

The steers were blocked by BW into three weight blocks, light, middle, and heavy, (*n* = 12, 24, and 12 pen replicates, respectively) based on d 0 BW, stratified by BW within block and assigned randomly to 1 of 48 pens. There were 10 steers/pen and 8 replications per treatment. Treatment design was a 2 × 3 factorial with 3 inclusions of CS (14%, 47%, and 80% of diet DM) with or without (+, −) the inclusion of a fumaric acid and palm oil additive (14 CS + FAPO, 14 CS − FAPO, 47 CS + FAPO, 47 CS − FAPO, 80 CS + FAPO, and 80 CS − FAPO; Table 1).

**Table 1.** Composition (% of diet DM) of dietary treatments fed to steers on varying inclusions of corn silage

	Treatment^*a*^					
	−FAPO			+FAPO		
Ingredient	14CS	47CS	80CS	14CS	47CS	80CS
Corn silage	14	47	80	14	47	80
Dry-rolled corn	66	33	-	66	33	-
Modified DGS	16	16	16	16	16	16
Supplement^*b*^						
Fine ground corn	1.2575	1.2575	1.2575	1.0575	1.0575	1.0575
Limestone	1.65	1.65	1.65	1.65	1.65	1.65
Urea	0.6	0.6	0.6	0.6	0.6	0.6
Salt	0.3	0.3	0.3	0.3	0.3	0.3
FAPO^*c*^	–	–	–	0.2	0.2	0.2
Tallow	0.1	0.1	0.1	0.1	0.1	0.1
Beef trace minerals premix	0.05	0.05	0.05	0.05	0.05	0.05
Rumensin^*d*^ premix	0.0165	0.0165	0.0165	0.0165	0.0165	0.0165
Vitamin A-D-E premix	0.015	0.015	0.015	0.015	0.015	0.015
Tylosin^*e*^ premix	0.011	0.011	0.011	0.011	0.011	0.011
Nutrient composition, % DM						
Organic matter	96.2	94.9	93.7	96.0	94.7	93.5
Neutral detergent fiber (aNDF^*f*^)	21.9	32.1	42.2	22.0	32.2	42.4
Crude protein	15.5	15.2	15.0	15.5	15.3	15.0
Ether extract	4.1	3.9	3.7	4.1	3.9	3.7

^*a*^CS, corn silage; FAPO, fumaric acid and palm oil additive (AL630US) contained palm oil, fumaric acid, and artificial flavors in calcium carbonate and sodium sulfate.

^*b*^Supplement fed at 4% of dietary DM for all treatments.

^*c*^Formulated to supply AL630US (Idena SAS; Saurton, France) at 0.2% of the diet DM.

^*d*^Formulated to supply monensin (Rumensin-90; Elanco Animal Health, Greenfield, IN) at 33.1 mg/kg.

^*e*^Formulated to supply tylosin (Tylan-40; Elanco Animal Health) at 9.7 mg/kg.

^*f*^aNDF: ash-free neutral detergent fiber.

Steers were started on the 80 CS diet and then adapted to 47 CS and 14 CS over a 10- and 24-d period, respectively, with dry-rolled corn replacing CS. Diets were formulated to meet or exceed NASEM (2016) requirements for protein, vitamins, and minerals. The +FAPO supplements were formulated, according to manufacturer recommendation, to supply 0.2% of the diet DM as FAPO (Idena SAS, Sautron, France). The FAPO (AL630US) contained palm oil, fumaric acid, and artificial flavors in calcium carbonate and sodium sulfate. Fumaric acid is a dicarboxylic acid and is a precursor to L-malate in the TCA cycle (National Center for Biotechnology Information 2019). Palm oil is the oil derived from the mesocarp of the fruit from oil palms (*Elaeis guineensis, Elaeis oleifera, Attalea maripa*; Reeves and Weihrauch 1979). The specific origin and inclusion level of the palm oil used in this study was not disclosed. The final finishing diets provided 330 mg/steer daily of monensin (Rumensin; Elanco Animal Health, Greenfield, IN), and 90 mg/steer daily of tylosin (Tylan; Elanco Animal Health). Tylosin and monensin were included in all diets, even with the inclusion of FAPO, to mimic common U.S. feeding practices. The most recent reports (2016) reported 87.5% of feedlots (regardless of size) used antimicrobials in feed, water, or by injection (USDA 2019). New regulations on antibiotics (medically important to humans, e.g., tylosin) have been implemented after these numbers were tabulated which may have lowered current percentages, but likely remain above 50%. Therefore, this study wanted to assess the effects of the addition of FAPO to typical U.S. diets that would include both monensin and tylosin. It is possible that because these additives alter the rumen environment and microbial populations, the FAPO used in this trial may respond differently than in diets with no monensin or tylsoin.

Steers were implanted with 200 mg of trenbolone acetate and 40 mg estradiol (Revalor-XS; Merck Animal Health, Madison, NJ) and received a bovine combination respiratory vaccine that helps in the prevention of BVD Type 1 virus, BVD Type 2 virus, BRSV, IBR virus and PI3 virus, and *Mannheimia haemolytica* (Bovi-Shield Gold One Shot; Zoetis Animal Health). All steers also received an injection to control gastrointestinal roundworms, lungworms, eyeworms, grubs, sucking lice, and mange mites with the addition of another injection to protect against *Histophilus somni*, a contributor to respiratory disease (Dectomax, Somubac; Zoetis Animal Health).

Shipping dates were calculated to target 1.27 cm of back fat. Cattle were fed for 238, 195, and 168 d for 80% CS, 47% CS, and 14% CS, respectively. These dates were determined based on back fat thickness using ultrasound scans. Four pens (one pen from each rep and each block) of cattle fed 47 CS treatment were ultrasounded on day 170 to compare with cattle harvested at 168 days (CS 14). Similarly, the same number of pens fed 80 CS were ultrasounded on days 170 to compare with the harvested cattle on day 168 (CS 14) and cattle fed CS 47. Additionally, cattle fed CS 80 were ultrasounded for a second time on day 198 to assess progress and compare with both cattle harvested at 168 days (CS 14) and 195 days (CS 47). Images were acquired using an Aloka SSD-500V (Hitachi Healthcare Americas) and were processed by The Cup Lab (Aimes, IA). Based on the differences among harvested animals (treatment groups), a regression of increasing back fat over days on feed was determined and the number of days until harvest was calculated (data not shown). Steers were shipped in the evening to a commercial abattoir (Greater Omaha Packing Co, Inc.; Omaha, NE) for harvest the next morning where carcass data were recorded. On day of harvest, hot carcass weight (HCW) was collected. Following a 48-h chill, USDA marbling score, longissimus muscle (LM) area, and 12th rib fat thickness were recorded. Carcass-adjusted performance was calculated using final body weight (FBW), based on HCW divided by a common dressing percentage of 63.8. Final body was calculated using HCW divided by a common (63.8%) dressing percentage. Average daily gain was calculated as the difference in initial BW and carcass adjusted FBW divided by the total days on feed.

Feed samples were collected weekly, weighed, and then dried in a 60°C forced-air oven for 48 h to determine DM content (AOAC, 1999; method 934.01). Dried feed samples were ground with a Wiley mill (Thomas Scientific, Swedesboro, NJ) through a 1-mm screen and composited by month. Ash and OM were measured by placing crucibles containing 0.5 g of each feed sample in a muffle furnace for 6 h at 600°C (AOAC, 1999; method 942.05). Crude protein was also analyzed using a combustion-type N analyzer (FlashSmart N/Protein Analyzer CE Elantech, Inc. Lakewood, NJ). Neutral detergent fiber analysis was conducted using the procedure described by Van Soest et al. (1991) with modifications to the analysis of corn and by-products described by Buckner et al. (2013). Additionally, 2 doses (0.5 mL/dose) of alpha amylase (Catalog # FAA, Ankom Technologies, Macedon, NY) were added during the hour-long reflux in ND solution. The modification applied to the by-products was a biphasic lipid extraction (Bremer et al. 2010) prior to NDF analysis (Buckner et al. 2013).

### CS Economics

CS inclusion was economically evaluated using a CS pricing application from Iowa State University (Edwards and Loy 2017). CS inclusion was economically evaluated using corn price based on market prices for September 2018 ($3.67). Dry corn price was calculated using $3.67 plus an average $0.20 (+ $0.05 per month on feed) with $2.17/ton DM charged for processing costs. Using the spreadsheet, CS price (maximum price to pay based on feed value) was calculated at $44.86 per metric ton DM ($121.30/metric ton as-is, 37% DM). The following inputs for expected production were 24.28 ha and 59.2 metric tons of silage (37% DM) per hectare (based on expected corn yield with 6% yield drag). The opportunity cost of harvesting and selling corn stover ($55.11/metric ton) as well as the cost to replace phosphate ($0.75/kg phosphate fertilizer) and potash ($0.55/kg potash fertilizer) after stalk removal was subtracted. Total replacement was estimated at 1.59 kg phosphate/unit of yield and 4.1 kg potash/unit of yield. Harvest and storage costs included $94.44/hectare for harvesting using a forage harvester and $0.11/metric ton for hauling and storing, accounting for 15% shrink loss. A credit was given for manure value. Manure credit was assessed as spreading 1 out of every 4 years in a rotation to provide enough phosphorus for 4 yr. This accounted for the cost of $3.95/metric ton of spreading manure every 4 yr. The value of manure was calculated using The Beef Feed Nutrient Management Planning Economics (BFNMP$) tool using 45% silage-based diet with 20% WDGS, adding up to a total value of $12.48/metric ton of silage intake (Koelsch 2013).

Cattle interest costs were set at 7.5% of the initial purchase price over the feeding period (Days on feed/365) including a $200 deposit. Corn price was based on market prices for September ($3.67/25.4 kg) with $2.39/metric ton DM charged for processing costs. The cost of MDGS was set at 90% the price of corn (DM basis) including 5% shrink. Supplement including monensin and tylosin was $331/metric ton (DM basis) with 1% shrink applied. Feed interest of 7.5% was applied to half of the total feed amount to average total usage throughout the feeding period. Medicinal and processing charges were $20/animal and yardage were charged to $0.50/animal / day. Feeder cattle price ($1.8382/50.8 kg) was calculated to target a net return of $0/animal for cattle on the 14% silage treatment. Returns were calculated as the difference in gross inputs and revenues where values represented profit in dollars per animal ($ / animal) and were calculated using FBW with a 63.8% common dressing percent to calculate live final weight and 5-year average (May 2014–May 2019) live fed price for Nebraska ($1.3055/50.8 kg; Livestock Marketing Information Center [LMIC], 2020).

Data from the last 5 years (May 2014–May 2019) had a correlation (*r*^2^ = 0.56) between feeder price and fed cattle (Livestock Marketing Information Center; LMIC). Lower correlation was observed in the last 5 years between feeder price and corn price (*r*^2^ = 0.35). However, historically, corn price and feeder calf price have been inversely related. A sensitivity analysis was conducted to assess the changes in returns based on changing corn price and feeder calf price. CS prices changed with the price of corn using the September market price. CS price compared with $3.00, $4.00, and $5.00/25.4 kg corn was $37.18 (per metirc ton DM), $45.00, $52.82, respectively. Feeder calf price was set to breakeven at 14 CS inclusion.

### Statistical Analysis

Carcass and performance data were analyzed as a generalized randomized block design using the MIXED procedure of SAS (SAS Institute, Inc. Cary, NC) where pen was the experimental unit. BW block (light, middle, or heavy) was considered a fixed effect. The experiment was analyzed as a 2 × 3 factorial with three inclusions of CS (14%, 47%, and 80% DM) and with or without FAPO. The effect of silage inclusion, the effect of FAPO inclusion, and the interaction between the two inclusions were analyzed as fixed effects. Two pens were removed from the data set because of cattle mixing. These pens were designated as missing data during statistical analysis. Treatment differences were declared significant for all statistical analysis at *P* ≤ 0.05.

## RESULTS AND DISCUSSION

All cattle were reached a common back fat of 1.29 cm (*P* = 0.95), when targeted to 1.27 cm, to ensure equal degree of finish when comparing performance and carcass characteristics. There was not a significant interaction (*P* ≥ 0.60) between the inclusion of FAPO in the diet and the inclusion level of silage for FBW, DMI, ADG, and G:F (Table 2). There were also no interactions for HCW, LM area, 12th rib fat, marbling, or calculated yield grade (*P* ≥ 0.15). Therefore, main effects for the FAPO and CS inclusion are presented.

**Table 2.** Carcass adjusted performance of cattle fed three inclusions of corn silage with or without a fumaric acid and palm oil additive

	Treatment^*^									
	14CS		47CS		80CS			*P*-value^*†*^		
	+FAPO	−FAPO	+FAPO	−FAPO	+FAPO	−FAPO	SEM	FAPO × CS	FAPO	CS
Pens, *n*	8	8	8	7	7	8	**–**	**–**	**–**	**–**
Days on feed	168	168	195	195	238	238	**–**	**–**	**–**	**–**
Feedlot performance^*‡*^										
Initial BW, kg	296	296	296	296	296	296	0.22	0.60	0.49	0.32
Final BW, kg	575	575	585	588	625	625	3.77	0.89	0.74	<0.01
DMI, kg/d	10.1	10.2	10.3	10.3	10.3	10.3	0.1	0.82	0.94	0.96
ADG, kg	1.66	1.66	1.48	1.50	1.38	1.38	0.02	0.88	0.76	<0.01
G:F	0.162	0.161	0.144	0.145	0.135	0.134	0.002	0.85	0.63	<0.01
NEm, Mcal/kg	1.87	1.88	1.74	1.75	1.68	1.69	0.13	0.99	0.29	<0.01
NEg, Mcal/kg	1.23	1.24	1.12	1.13	1.06	1.08	0.01	0.87	0.33	<0.01
Return, $/animal	−2.75	0.74	−8.08	−2.46	43.98	42.28	8.24	0.83	0.98	<0.01
Carcass characteristics										
HCW, kg	367^c^	367^c^	373^bc^	375^b^	399^a^	398^a^	2.39	0.88	0.72	<0.01
LM area, cm^2^	85	83.8	82.6	83.9	81.3	83.9	0.9	0.55	0.04	0.11
12th rib fat, cm	1.26	1.31	1.28	1.29	1.27	1.32	0.04	0.59	0.99	0.98
Marbling^*||*^	467	468	433	461	459	469	10.5	0.32	0.13	0.1
Calculated yield grade^*$*^	3.24^a^	3.29^a^	3.01^b^	3.07^b^	3.25^a^	3.29^a^	0.04	0.15	0.79	<0.01

^abc^Means within a row without common superscript are significantly different (*P* < 0.05).

^*^CS, corn silage; FAPO, fumaric acid and palm oil additive (AL630US, Idena SAS; Soutron France) contained palm oil, fumaric acid, and artificial flavors in calcium carbonate and sodium sulfate.

^*†*^FAPO × CS = *P*-value for the interaction between corn silage inclusion and FAPO inclusions; FAPO = *P*-value for the main effect of FAPO inclusion; CS = *P*-value for the main effect of corn silage inclusion.

^*‡*^Calculated on a carcass-adjusted basis using a common dressing percentage (63.8%).

^*||*^Marbling Score 300 = Slight, 400 = Small, 500 = Modest, etc.

^*$*^Calculated YG = 2.50 + (6.35 × fat thickness, cm) + (0.2 × KPH, %) + (0.0017 × HCW, kg) − (2.06 × LM area, cm^2^).

### Fumaric Acid and Palm Oil Additive Effects

There were no significant differences (*P* ≥ 0.49) due to the inclusion of FAPO for carcass adjusted animal performance including FBW, DMI, ADG, and G:F (Table 3). Benchaar et al. (2006b, 2007) observed no differences in DM intake, milk production, and milk components when dairy cows were fed 750 mg or 2 g of FAPO daily. Benchaar et al. (2006a) evaluated growth performance of beef cattle fed a 75% grass silage and 24% rolled barley diet supplemented with 2 or 4 g/day of EO compounds (Vertan, IDENA, Sautron, France) consisting of thymol, eugenol, vanillin, and limonene. The author’s results showed that DMI and ADG were not affected by the inclusion of EO. However, feed efficiency had a quadratic effect with 2 g/day of EO yielding maximum feed efficiency. Notably in the same study there was an interaction for DMI to be greatest when EO was fed with monensin. Similarly, Pukrop et al. (2017) found no difference due to EO (1 g/d; origin not reported) for performance characteristics, including weight, days on feed, ADG, DMI, and G:F. No differences were observed in carcass characteristics (*P* ≥ 0.19). Another study with goats found no difference in feed efficiency, growth performance or intake when fed 0%, 4% (34 g/d), or 8% (68.5 g/d) blend of 80% canola oil and 20% palm oil (Adeyemi et al. 2016). Beauchemin and McGinn (2006) fed 29 g/kg DMI (175 g/d) of fumaric acid in a 75% barley silage diet and found no difference in DMI or ADG. Choi et al. (2013) found no difference in ADG or G:F when cattle were fed 3% palm oil compared with a control.

**Table 3.** Main effect of fumaric acid and palm oil additive on carcass adjusted performance with cattle fed three inclusions of corn silage

	Treatment^*a*^			
	+FAPO	−FAPO	SEM	*F*-test
Pens, *n*	23	23	–	–
Days on feed	200	200	–	–
Feedlot performance^*b*^				
Initial BW, kg	296	296	0.12	0.49
Final BW^2^, kg	595	590	2.09	0.74
DMI, kg/d	10.3	10.3	0.05	0.94
ADG, kg	1.50	1.51	0.01	0.76
G:F	0.147	0.147	0.001	0.66
NEm Mcal/kg	1.77	1.76	0.007	0.29
NEg Mcal/kg	1.15	1.14	0.006	0.33
Return, $/animal	12.86	12.69	4.63	0.98
Carcass characteristics				
HCW, kg	380	380	1.4	0.72
LM area, cm^2^	82.6	84.5	0.5	0.04
12th rib fat, cm	1.285	1.285	0.02	0.99
Marbling^*c*^	453	466	5.9	0.13
Calculated yield grade^*d*^	3.18	3.20	0.02	0.61

^*a*^FAPO, Fumaric acid and palm oil additive (AL630US, Idena SAS; Soutron, France) contained palm oil, fumaric acid, and artificial flavors in calcium carbonate and sodium sulfate.

^*b*^Calculated on a carcass-adjusted basis using a common dressing percentage (63.8%).

^*c*^Marbling Score 300 = Slight, 400 = Small, 500 = Modest, etc.

Calculated YG = 2.50 + (6.35 × fat thickness, cm) + (0.2 × KPH, %) + (0.0017 × HCW, kg) − (2.06 × LM area, cm^2^).

The FAPO in this study included palm oil. A study by Meyer et al. (2009) showed no difference in carcass characteristics (HCW, fat thickness, LM area, and marbling) when cattle were fed an 82.5% concentrate diet with an EO mixture (1 g/d; thymol, eugenol, vanillin, guaiacol, and limonene) plus tylosin compared with cattle fed only monensin and tylosin. Similarly, Pukrop et al. (2017) found no differences in carcass characteristics when EO (origin not reported) was included at 1 g/steer daily in a diet of 54% cracked corn, 14% DDGS, and 14% CS. Choi et al. (2013) found slightly greater marbling scores when cattle were fed 3% palm oil (33 g/d) compared with a control (*P* < 0.09). In a digestibility study by Benchaar et al. (2006a), no interaction for DM, OM, CP, NDF, or ADF digestibility were observed when dairy cows were fed a combination of EO (1 g/d; thymol, eugenol, vanillin, and limonene) and monensin. This suggests that even with the inclusion of tylosin and monensin in the current study, results likely would not have been altered due many studies observing no interaction between the inclusion of monensin and tylosin with the inclusion of EO.

Fumaric acid was an additive in the current studies FAPO additive. Fumaric acid has been identified as a metabolic precursor to propionate (Itabashi 2002). It has been reported that the influence of fumaric acid depends on the roughage used in a diet (Remling et al. 2017). McGinn et al. (2004) fed cattle 10.6 g/kg DMI (80 g/d) of fumaric acid in a 75% barley silage diet and found no effect on total VFA concentration or the proportion of propionate. Similarly, Beauchemin and McGinn (2006) fed 29 g/kg DMI (175 g/d) of fumaric acid in a 75% barley silage diet and found a decrease in acetate:propionate ratio but no effect on methane production. Conversely, Bayaru et al. (2001) fed 20 g/kg DMI of fumaric acid in a 75% sorghum silage diet and reported a 20% reduction in methane when fed to cattle. Similarly, a digestion study with goats found an increased ruminal pH and a decrease in total VFA concentration and methane production when a 37% ground corn, 23% alfalfa hay diet, was supplemented with 24 g/d of fumaric acid (Li et al. 2018). However, the magnitude of methane mitigation was greater in a diet with a slowly fermentable corn source compared with a highly fermentable corn source. Abubakr et al. (2014) observed a significant decrease in ruminal methanogenic archea when goats were fed 5% palm oil added to a 60% corn grain, 10% rice straw, and 22% soybean meal diet (% DM).

Total VFA concentration is an indicator of ruminal fermentation (Cardozo et al. 2005). Lowering the ratio between acetate:propionate shifts fermentation to be more efficient for beef production systems (Wolin et al. 1997). Studies have observed a relationship where VFA concentrations are reduced when high doses of various EO are fed (Cowan 1999; Evans and Martin 2000; Busquet et al. 2004). However, no interactions between monensin and EO (thymol, eugenol, vanillin, and limonene) were observed for VFA concentrations, pH, or protozoa counts in dairy cows (Benchaar et al. 2006a).

There are many different EO and plant extracts that have varying effects on ruminal fermentation and digestion (Kamra et al. 2005). Moreover, the effects of diet composition add to the complexity of these interactions (Calsamiglia et al. 2007; Cobellis et al. 2016). A study by Cardozo et al. (2005) found no potential benefit to ruminal fermentation by dosing plant extracts, especially in a beef production setting where diets tend to yield high ruminal pH like forage based diets, compared with lower pH in cattle fed high concentrate diets. Supplementing 400–3,000 mg/L of various plant extracts had negative impacts on VFA production at a pH of 7.0. Lower doses of 3–30 mg/L showed no negative effects on total VFA concentration. The authors attributed the insignificant response to low doses of plant extracts not being toxic to the ruminal microbes. However, when the same experiments were conducted at a pH of 5.0 some extracts (eugenol, garlic, capsicum, cinnamaldehyde, and yucca) yielded greater total VFA concentrations and a lower acetate:propionate ratio, while others remained similar to the control (antehole, anise, oregano, cinnamon). Another study done by Carro and Ranilla (2003) described that adding fumarate to concentrate feed cultures increased VFA concentrations, decreased acetate:propionate ratio, and decreased methane production. Other in vitro studies have reported similar findings with decreased methane production and increased propionate concentrations (Asanuma et al. 1999;Lopez et al. 1999). This shift in fermentation profile has been commonly found in methane inhibitors (Chalupa et al. 1980; Martin and Macy 1985; Busquet et al. 2005). In combination with the accumulation of ammonia nitrogen concentrations, it has been suggested that EO extracts stimulate deamination of amino acids which could be beneficial in feedlot cattle fed high percentages of concentrate where microbial protein synthesis is limiting (Cardozo et al. 2005). A study by Abubakr et al. (2014) found a significant decrease in ruminal methanogenic archaea when goats were fed 5% palm oil added to a 60% corn grain, 10% rice straw, and 22% soybean meal diet (% DM).

Suggested mechanisms for the use of EO are related to the ability of the compounds to disrupt the cell membrane of bacteria (Griffin et al. 1999; Dorman and Deans 2000; Ultee et al. 2002). Many EO may decrease proton movement across the cell membrane causing a subsequent decrease in ATP production which ultimately slows microbial growth (Ultee et al. 2002). However, different EO may have other mechanisms that act on the inner organelles of the microbial cell (Helander et al. 1998; Busquet et al. 2005). Thus, the overall effects of EO on rumen microbial fermentation most likely depend on specific microbial populations and their sensitivities to certain compounds in which ruminal pH plays a critical role (Cardozo et al. 2005). Some short-chain organic acids (C1–C7) have antimicrobial activity when the pKa value is from 3 to 5 (Papatsiros et al. 2013). Fumaric acid (C_4_H_4_0_4_), used in this study, has a pKa of 3.03 suggesting it may have antimicrobial properties. Fumaric aid given at 24 g/d reduced the abundance of methanogens in goats fed both low and high concentrate diets by 3 × 10^–4^ (Li et al. 2018). A study using palm oil extract reported antibacterial activity against *Staphylococcus aureus* and *Escherichia coli* (Febrina et al. 2018). Additionally, reduction in protozoa and methanogens in the rumens of goats was reported by Abubakr et al. (2014) when goats were fed 5% palm oil. However, there were no effects of FAPO observed in this study perhaps suggesting no shift in rumen microbial fermentation and no significant antimicrobial effects in these specific diets.

### CS Effects

By design, all cattle were targeted to reach 1.27 cm of back fat. Actual measured back fat was the same for all inclusion of CS (1.29 cm; *P* = 0.98; Table 4) which required different days on feed. There was a significant difference in FBW with silage inclusion affecting BW in a linear and quadratic manner (*P* < 0.01). Cattle fed 80 CS had the greatest FBW, followed by 47 CS, and least for 14 CS. Dry matter intake was not significantly different for the three silage inclusions (*P* = 0.96). There was a linear and quadratic response for ADG and G:F (*P* ≤ 0.04). Cattle fed 14 CS had the greatest ADG and G:F ratio, followed by 47 CS, and least for 80 CS. These results agree with data where increasing concentration of CS resulted in cattle performance with lower ADG and poorer feed efficiencies (Hammes et al. 1964; Klosterman et al. 1965; Jesse et al. 1976; Brennan et al. 1987). Erickson (2001) evaluated CS in finishing diets at 15%, 30%, and 45% inclusions. There were no differences in DMI when these inclusions were fed to yearling cattle. However, DMI increased as CS inclusion increased when fed to calf feds. In both groups, ADG and G:F linearly decreased with increase inclusion of CS. Hilscher et al. (2019) reported a decrease in ADG when CS inclusion was increased from 15% to 45% of diet DM, but no difference in DMI. A study by Peterson et al. (1973) evaluated cattle fed inclusions of CS at 28.6%, 57.1%, and 85.7% and cattle were fed a range of 172–227 days on feed. The authors reported a linear decrease in ADG and G:F as CS inclusion increased. Similarly, Gill et al. (1976) fed 14%, 30%, or 75% CS and found lower ADG and poorer feed conversions for cattle fed 75% CS. It is important to note that cattle on the current study were fed to a common back fat, where the previously mentioned studies fed cattle the same number of days.

**Table 4.** Main effect of corn silage on carcass adjusted performance of cattle fed three inclusions of corn silage

	Treatment^*^						
	14CS	47CS	80CS	SEM	*F*-Test	Linear	Quadratic
Pens, *n*	16	15	15	–	–	–	–
Days on feed	168	195	238	–	–	–	–
Feedlot performance^*†*^							
Initial BW, kg	296	296	296	0.15	0.32	0.22	0.37
Final BW, kg	575^c^	586^b^	625^a^	2.59	<0.01	<0.01	<0.01
DMI, kg/d	10.3	10.3	10.3	0.07	0.96	0.85	0.84
ADG, kg	1.66^a^	1.49^b^	1.38^c^	0.01	<0.01	<0.01	0.04
G:F	0.161^a^	0.145^b^	0.134^c^	0.001	<0.01	<0.01	<0.01
NEm Mcal/kg	1.87	1.75	1.69	0.009	<0.01	<0.01	<0.01
NEg Mcal/kg	1.23	1.12	1.07	0.007	<0.01	<0.01	<0.01
Return, $/animal	−1.09^a^	−6.35^a^	45.76^b^	5.68	<0.01	<0.01	<0.01
Carcass characteristics							
HCW, kg	367^c^	374^b^	399^a^	1.6	<0.01	<0.01	<0.01
LM area, cm^2^	84.5^a^	83.2^ab^	82.6^b^	0.65	0.11	0.04	0.61
12th rib fat, cm	1.29	1.28	1.29	0.03	0.98	0.87	0.90
Marbling^*‡*^	468^ab^	447^c^	464^bc^	7.30	0.1	0.71	0.04
Calculated yield grade^*||*^	3.27^a^	3.04^b^	3.27^a^	0.03	<0.01	0.88	<0.01

^abc^Means within a row without common superscript are significantly different (*P* < 0.05).

^*^CS = corn silage.

^*†*^Calculated on a carcass-adjusted basis using a common dressing percentage (63.8%).

^*‡*^Marbling Score 300 = Slight, 400 = Small, 500 = Modest, etc.

^*||*^Calculated YG = 2.50 + (6.35 × fat thickness, cm) + (0.2 × KPH, %) + (0.0017 × HCW, kg) − (2.06 × LM area, cm^2^).

In the current study, there was a linear and quadratic response for HCW (*P* < 0.01). Cattle fed 80 CS had the greatest HCW, followed by 47 CS, and least for 14 CS. There was a linear response for LM area (*P* = 0.04) where cattle fed 14 CS had the greatest LM area, 47 CS was intermediate, and least for 80% CS. Marbling score was quadratic (*P* = 0.04) with cattle fed 14 CS having the greatest marbling score, 80 CS was intermediate but similar to 14 CS, and 47 CS was least. Hilscher et al. (2019) reported no difference in LM area or marbling score when cattle were fed 15% or 45% CS, but observed lower final BW for cattle fed 45% CS when cattle were fed the same number of days.

### CS Economics

The inclusion of FAPO did not impact returns (*P* = 0.98; Table 3). Greater returns were projected as CS inclusion increased (*P* < 0.01; Table 4) but the extent of returns was dependent on the price relationships for feed and steer prices. Projected profitability was least (−$6.35/animal) for feeding 47 CS. This may be due to longer days on feed relative to the to the steer’s ADG and fat deposition. The greatest profitability projected came from cattle fed 80 CS throughout the feeding period. The economically favorable price of CS compared with DRC ultimately led to the large increase in returns when steers were priced over $2.64 per 0.45 kg.

CS has previously been shown to be economical in times of expensive corn while adding utility to cattle producers by securing substantial inventories of roughage and grain (Goodrich et al. 1974; Gill et al. 1976; DiCostanzo et al. 1998). In this study the price of calves and the price of corn impacted gross revenue of the system equally with corn representing a large proportion of overall feed costs which concurrently impacted CS pricing. Evaluating the response in profitability to associated changes in both steer and corn price provides the most information regarding economic feasibility of feeding different levels of CS when cattle are finished to a common fatness. Because feed costs heavily influence profitability, differences in returns ($/animal), based on corn price, were evaluated at the varying inclusions of CS. As corn (and CS) price increased, there was a greater difference in the returns ($/animal) when cattle were fed 80 CS. For example (Table 5), at $3.00 corn, cattle fed 80 CS returned an additional $41.99 per animal compared with cattle fed 14 CS. Furthermore, when corn was $5.00, returns were ($59.76/animal) greater for cattle fed 80 CS compared with 14 CS. Cattle fed 47 CS did not perform as expected. However, returns became greater than feeding at 14 CS when corn was $4/25.4 kg or above. The same trend held true where increasing corn price led to an increase in returns as $/animal. These data suggest, as corn becomes more expensive, it becomes more economical to feed CS at greater inclusions.

**Table 5.** Estimated returns ($/animal) at varying corn prices for three inclusions of corn silage fed to feedlot cattle^*a*^

	Treatment^*b*^			
Dry corn price^*c*^, $/25.4 kg	Feeder calf price^*d*^, $/50.8 kg	14 CS	47 CS	80 CS
3.00	1.9313	0.03	−5.86	42.02
4.00	1.8243	0.01	2.07	50.86
5.00	1.7172	0.06	1.77	59.76

^*a*^Returns calculated as the difference in gross inputs and revenues. Values represent profit in dollars per animal ($/animal). Inputs: Total feed costs including processing and shrink. Cattle interest = ([days on feed/365] × [feeder price − $200] × 0.075). Feed interest = ([Total feed costs/2] × 0.075 × [days on feed/365]). Yardage = $ 0.50/animal/d. Processing = $20/animal. Revenue: FBWs using a 63.8% common dressing percent to calculate live final weight and 5-year average live fat cattle price for Nebraska ($1.3055/50.8 kg).

^*b*^CS = corn silage.

^*c*^Corn silage prices floated with the price of corn utilizing a September corn price comparison ($−0.20/25.4 kg) compared with $3/25.4 kg, $4, and $5 dry corn. The corn silage prices were $37.18 (per 909 kg DM), $45.00, $52.82, respectively.

^*d*^Initial purchase price of feeder calves was set to break even for 14CS treatment.

Because there are different effects for every EO it may be difficult to draw conclusions from individual studies. In this study, inclusion of palm oil and fumaric acid did not affect animal performance in diets containing monensin and tylosin. The inclusion of FAPO had no effect on performance, carcass quality or profitability. Greater inclusions of CS decreased ADG and G:F but led to greater FBWs when finished to a common back fat thickness. Additionally, greater inclusions of CS led to increased profitability in dollars per animal sold. Feeding CS with or without the inclusion of FAPO is an economical system to finish cattle.
